# Diving in the big blue: Atypical Mott cells in rapidly fatal primary amyloidosis

**DOI:** 10.1002/jha2.582

**Published:** 2022-10-06

**Authors:** Kenza Rwayane, Baptiste Le Calvez, Valentin Le Roc'h, Clara Sortais, Marion Eveillard

**Affiliations:** ^1^ Hematology Biology Nantes University Hospital Nantes France; ^2^ Pediatric Oncology Nantes University Hospital Nantes France; ^3^ Hematology Department Nantes University Hospital Nantes France; ^4^ Nantes Université, INSERM, CNRS Université d'Angers, CRCI2NA Nantes France

**Keywords:** amyloidosis, mott cell, plasma cell

1

A 75‐year‐old man with no previous medical history presented at the emergency unit with asthenia, anorexia, weight loss, and progressively increasing dyspnea revealing acute heart failure (N‐terminal pro b‐type natriuretic peptide:16 667 ng/L; troponemia: 47 ng/L). Echocardiography showed a granular appearance of the myocardium and interventricular septal hypertrophy, suggestive of amyloidosis. Chemistry disclosed high levels of immunoglobulin kappa light chains at 1240 mg/L (lambda light chains 14 mg/L; κ/λ ratio 86) and a detectable but unquantifiable IgA κ peak. Myocardial biopsies pathology showed an endocardium thickened by fibrosis. Within the myocardium, the presence of interstitial fibrillary deposits stained by Congo red and birefringent in polarized light confirmed the diagnosis of cardiac amyloid light‐chain (AL) amyloidosis. The search for other organ damage disclosed vegetative involvement with orthostatic hypotension, icteric cholestasis (bilirubin 51.8 mg/L), undocumented digestive involvement (diarrhea with evocative endoscopic lesions), and glomerulonephritis (urinary proteinuria 4 g/24 h with 3 g/24 h albumin and 1 g/24 h immunoglobulin light chains).

A bone marrow aspirate displayed 17% of plasma cells. Interestingly, a particular pattern of huge atypical Mott cells was identified (Figure 1A,B). The latter had so many cytoplasmic inclusions that the cytoplasm, and the nucleus could not be discriminated. These atypical cells are however plasma cells with an endoplasmic reticulum loaded with immunoglobulins and should be integrated as such in cell counts. Classical plasma cells and typical Mott cells with a few Russel bodies were also present (Figure 1C) indicating a continuum in the shape of the diseased plasma cells.

**FIGURE 1:**
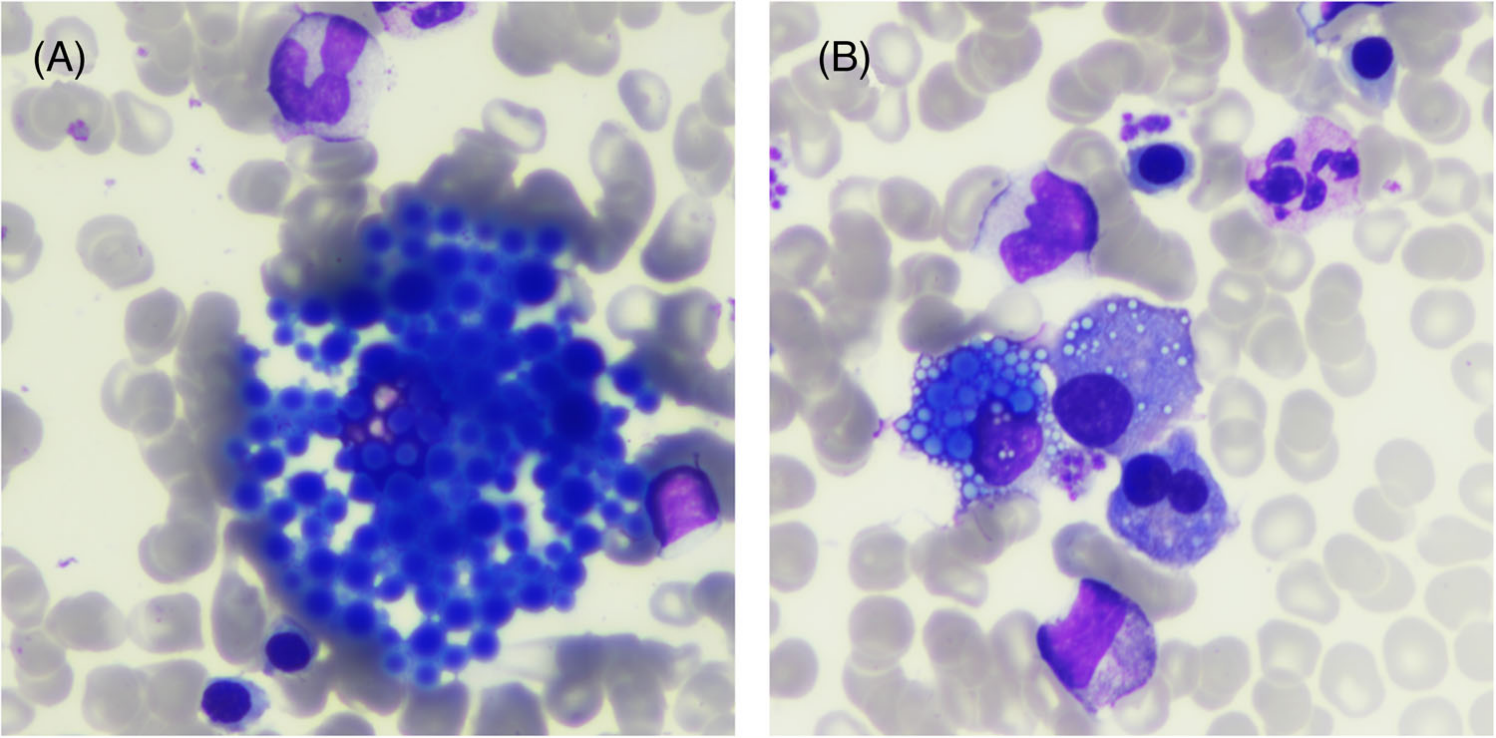
Bone marrow aspiration with classical and atypical Mott Cells. (A and B) Objective ×50

Treatment with cyclophosphamide–bortezomib–dexamethasone‐daratumumab was initiated in emergency. Unfortunately, the patient died 3 days later, of multiple organ failure. No additional septic or toxic causes could be identified.

Mott cells are activated plasma cells containing intracytoplasmic inclusions with a “bunch of grapes” appearance. These inclusions, called Russell bodies, are dilated cisternae of the endoplasmic reticulum containing condensed immunoglobulins. They were identified in 1901 by the surgeon F.W Mott in the brains of monkeys with trypanosomiasis. Mott cells have been described in various pathological conditions: reactive plasmacytosis, monoclonal gammopathies, high grade B‐cell lymphomas, Wiskott‐Aldrich syndrome, and von Recklinghausen neurofibromatosis.

AL amyloidosis is caused by clonal expansion of plasma cells producing misfolded immunoglobulin light chains responsible for the deposition of insoluble fibrillar proteins in many organs. Those most frequently affected are the kidneys, heart, liver, and peripheral nervous system. AL amyloidosis is a rare disease, and its diagnosis is often delayed. Although associated with a poor prognosis, AL amyloidosis presents a heterogeneous clinical expression and evolution. To our knowledge, the presence of Mott cells has rarely been described in this pathology.

## AUTHOR CONTRIBUTIONS

KR, ME, and BLC wrote the manuscript. KR, ME, and BLC performed biological analyses. CS treated the patient. All authors critically reviewed the manuscript.

## CONFLICT OF INTEREST

The authors have no conflict of interest to disclose.

## FUNDING INFORMATION

The authors received no specific funding for this work.

## ETHICS STATEMENT

The research was conducted in accordance with the principles embodied in the Declaration of Helsinki and in accordance with local statutory requirements.

